# Cognitive-reminiscence therapy and usual care for depression in young adults: study protocol for a randomized controlled trial

**DOI:** 10.1186/1745-6215-14-343

**Published:** 2013-10-21

**Authors:** David J Hallford, David Mellor

**Affiliations:** 1 School of Psychology, Deakin University, 221 Burwood Hwy, Burwood, VIC 3125, Australia

**Keywords:** Cognitive-reminiscence therapy, Depression, Reminiscence therapy, Young adults

## Abstract

**Background:**

Depression is a common affliction for young adults, and is associated with a range of adverse outcomes. Cognitive-reminiscence therapy is a brief, structured intervention that has been shown to be highly effective for reducing depressive symptoms, yet to date has not been evaluated in young adult populations. Given its basis in theory-guided reminiscence-based therapy, and incorporation of effective therapeutic techniques drawn from cognitive therapy and problem-solving frameworks, it is hypothesized to be effective in treating depression in this age group.

**Methods and design:**

This article presents the design of a randomized controlled trial implemented in a community-based youth mental health service to compare cognitive-reminiscence therapy with usual care for the treatment of depressive symptoms in young adults. Participants in the cognitive-reminiscence group will receive six sessions of weekly, individual psychotherapy, whilst participants in the usual-care group will receive support from the youth mental health service according to usual procedures. A between-within repeated-measures design will be used to evaluate changes in self-reported outcome measures of depressive symptoms, psychological wellbeing and anxiety across baseline, three weeks into the intervention, post-intervention, one month post-intervention and three months post-intervention. Interviews will also be conducted with participants from the cognitive-reminiscence group to collect information about their experience receiving the intervention, and the process underlying any changes that occur.

**Discussion:**

This study will determine whether a therapeutic approach to depression that has been shown to be effective in older adult populations is also effective for young adults. The expected outcome of this study is the validation of a brief, evidence-based, manualized treatment for young adults with depressive symptoms.

**Trial registration:**

Australian New Zealand Clinical Trials Registry ACTRN12613000084785.

## Background

Depression is a prevalent mood disorder that is one of the leading causes of disability worldwide [1]. Although depression affects individuals across the adult lifespan, onset typically occurs in adolescence or young adulthood [2], with the average age of first episode around 25 years [3,4]. The early onset of depressive symptoms, prior to the age of 25, has also been found to be predictive of a longer duration of symptoms, increased number of depressive episodes and higher frequency of suicide attempts relative to later onset of depression (over 40 years of age) [5]. In Australia, findings from the 2007 National Survey of Mental Health and Wellbeing [6] indicated the 12-month prevalence of major depressive episodes to be 4.1%. However, for individuals aged 16 to 24, this prevalence was higher, at 6.3%. Furthermore, some evidence suggests that this prevalence is increasing [7].

Psychological therapies for depression can provide long-term positive outcomes for sufferers of depression [8,9] and reduce the risk of further episodes [10-12]. However, a substantial number of individuals do not respond to currently available psychological treatments [13], and expert working groups have recommended the continued development or refinement of existing therapies [14]. It is therefore clear that there exists an imperative for the evaluation of new interventions for treating depressive symptoms in young adults.

Reminiscence-based psychotherapies, which utilize the purposeful review and analysis of autobiographical memories as a core component of therapy, have been shown to be an evidence-based treatment approach for attenuating depressive symptoms [15]. An extensive body of research investigating the associations between memory and depression indicates that reminiscence plays an important role in the maintenance of symptoms. Compared with those who are not depressed, clinically depressed individuals remember relatively more emotionally negative events [16] and use a more depressive explanatory style when recalling life narratives [17]. Depressed individuals also exhibit a tendency for overgeneralization when recalling memories, and this can impair their ability to cope with stressors through problem solving and reduce their ability to use memories to predict future events rationally and accurately. Furthermore, it is predictive of more severe depressive symptoms and delayed recovery [18,19].

Meta-analyses of evaluations of reminiscence-based therapies [15,20] have shown significant effects for these approaches in reducing depressive symptoms. Those reminiscence-based therapies that incorporate other therapeutic frameworks for conceptualizing and treating depression have been particularly compelling. Of these, cognitive-reminiscence therapy has demonstrated perhaps the most promising outcomes, with consistently large pre-post-treatment effect sizes [21-23] and high patient-reported satisfaction [23]. Cognitive-reminiscence therapy incorporates structured and guided reminiscence utilizing a Beckian cognitive therapy model of depression treatment [24,25] and a stress and coping model of depression [26,27] with problem-solving techniques [28]. Although this treatment has only been evaluated with older adults, it has been argued that it may also be appropriate and effective in young adult populations [29].

In cognitive-reminiscence therapy, therapists assist individuals in reviewing events and experiences from their life whilst using established therapeutic techniques from the frameworks discussed previously to restructure negative and dysfunctional beliefs, positively reframe and re-story narratives, and build self-efficacy and coping skills. It is proposed that this approach works through several specific mechanisms to treat depression. Firstly, increasing autobiographical memory specificity can disrupt depressed individuals’ tendency to use overgeneral and negatively-valanced memories. Indeed, results from several recent trials have shown that simply eliciting specific autobiographical memories in a concerted manner can decrease depressive symptoms [30,31] and increase problem-solving skills [32]. Secondly, the cognitive therapy techniques employed when reviewing past memories, such as psychological distancing and adaptively restructuring beliefs, have been shown to underpin reductions in depressive symptoms [33,34]. Thirdly, the problem-solving techniques that are used to highlight past coping ability, build a sense of resilience and formulate new solutions to current challenges, have been shown to be effective for treating depression [28], and specifically for young adults [35].

Previous trials of cognitive-reminiscence therapy have examined its effects in a group-based format, typically with two to four individuals [21-23]. Little is currently known though about the effect of this intervention in a one-to-one, individual therapy format. Relative to group therapy, an individual format for treatment would conceivably allow the therapist and client additional time within sessions to examine and reinterpret events and experiences from the past more fully, to form a more positive life narrative. Research generally supports the notion that individual therapy is more effective than group therapy in the treatment of depression [36,37]; however, this premise has yet to be tested in the case of cognitive-reminiscence therapy.

In summary, cognitive-reminiscence therapy has been shown to be a highly effective intervention for depressive symptoms that has not been evaluated with young adults to date. Our trial will aim to rectify this gap in the clinical research literature by implementing a randomized, controlled trial to provide evidence of its effectiveness relative to usual care in a community-based youth mental health service. The trial was designed to address two questions. Firstly, is cognitive-reminiscence therapy effective in reducing depressive symptoms in young adults? And secondly, how effective is cognitive-reminiscence therapy in comparison with usual care at a youth mental health service?

## Methods and design

### Study design

To evaluate the effectiveness of cognitive-reminiscence therapy in the treatment of young adults with depressive symptoms, we will conduct a randomized controlled trial and compare this intervention with usual care. We will utilize a between-within, repeated-measures design over five time-points (baseline, three weeks into the intervention, post-intervention, one month post-intervention, and three months post-intervention) to assess for both within-group change and differences between these groups in change.

### Participants

Young adults will be recruited from a community youth mental health service in the city of Melbourne, Australia. As the eligible age of intake at these participating services is capped at 25 years of age, it is not anticipated that any individuals will exceed this age range. The inclusion and exclusion criteria for participation will be:

1. 18 years of age, or older.

2. The presence of at least moderate depressive symptoms, as indicated by a score of seven or higher on the DASS-21 depression subscale [38].

3. Not currently receiving any other treatment for depression.

4. Depressive symptoms are not the product of physical causes (for example, hypothyroidism).

5. Not at high risk of suicide or harm to others (as assessed through intake interviews).

6. Not presenting with a primary problem related to psychotic symptoms, disordered eating, manic symptoms, body image disturbance, anxiety, substance use or sexual dysfunction.

Individuals will only be excluded from participating in the study in the case that problems other than depression are the primary reason for presentation (as assessed at intake), or other problems emerge as the primary reason for help-seeking during treatment and therefore supersede depressive symptoms as the likely target for therapy (as assessed during the course of the study).

### Procedure

Initially, intake clinicians working at the youth mental services will identify individuals eligible to participate in the study through assessments conducted as part of their normal triage protocol. This includes a structured face-to-face assessment of psychosocial and mental health problems, which provides the information necessary to determine eligibility based on the criteria listed [39]. The study will be introduced to eligible individuals, who will then be screened for the presence of at least moderate depressive symptoms using the DASS-21 depression subscale [38] and provided with the plain language statement. Permission for a member of the research team to contact them will be sought. A member of the research team will then contact them to discuss the details of the study further and answer any questions they may have. If the individuals agree to participate, informed consent will be sought and participants will be randomized into one of the treatment conditions.

### Randomization

Participants will be randomized to conditions using an adaptive biased-coin approach, the ‘urn design’ [40]. In an urn design, the probability of assignment to a group begins at 1:1, and is subsequently based on the magnitude of imbalance between groups. If the allocation ratio is found to be too far in one group’s favour then the probability is altered to increase the odds of allocation to the smaller group until this imbalance is rectified. This approach was chosen so as to counteract the risk of unequal group sizes, whilst still preserving most of the unpredictability of simple randomization. Randomization will be performed by a third-party outside of the research team using the online software Research Randomizer Version 3.0 [41].

### Sample size

Sample size was calculated using GPower 3 [42], with two aims of the current study under consideration: whether participants in the cognitive-reminiscence therapy group report improvements on measures over time, and whether these effects differ from those achieved by usual care. Although previous evaluations of this intervention with older adults have produced large effect sizes; [21-23], it was decided to err on a somewhat more conservative side and estimate a sample size for a moderate-to-large within-group effect over time. It was calculated that using a repeated-measures within-subject analysis of variance (ANOVA) across five time-points with an α level set at 0.05 (two-tailed), statistical power level (1 − β) of 0.80, and an estimate of correlation among repeated measures of 0.5, 15 participants will be needed in the cognitive-reminiscence group to detect this effect. To assess for a small-to-moderate interaction effect between groups and time using the parameters described above, a total of 32 participants (16 per group) will be needed. After factoring in the average dropout rate of 20% for reminiscence-based therapies [20], it is estimated that a minimum of 19 participants per group (total *N* = 38) will need to be recruited to assess within-group effects and differences between groups in treatment effects.

### Conditions

#### Cognitive-reminiscence

Participants in this group will receive an intervention based on a manualized reminiscence therapy formulated by Watt and Cappeliez [21]. Although the intervention will follow the general protocol of this manual, several distinct adaptations will be made for the current study. Firstly, the original manual described separate but overlapping protocols for integrative and instrumental reminiscence therapy. The current intervention will combine these two approaches, as per Cappeliez [23]. Secondly, the content of the sessions will be organized on the basis of only one participant, rather than a group delivery, for which it was originally designed. Thirdly, the questions used in sessions and on homework sheets to elicit memories will be age-appropriate for young adults and their stage of psychosocial development.

The intervention will be administered on the premises of the participating youth mental health service. The intervention comprises six 60-minute weekly sessions of individual psychotherapy. Over the course of therapy, six different topics are used to stimulate memories as a basis for therapeutic work. These are: turning points, family life, major life works, loves and hates, stressful experiences, and meaning in life. In the first session, rapport is built and psychoeducation is provided on autobiographical memory (specifically, integrative and instrumental types of reminiscence), related depressogenic patterns, and cognitive therapy and stress and coping models of depression. Information is provided about the intervention, including evidence for its effectiveness and what it entails for the participant and therapist. Following this, the first topic, turning points, is discussed, using a list of questions to help stimulate memories. The procedure for therapeutic work then follows the same pattern in each session. Memories are elicited and among these several are identified by the therapist and participant collaboratively as being of importance and to be discussed. The therapist ensures that there is at least one event or experience that is likely to benefit from cognitive therapy techniques and one that relates to stress and coping. These memories are then elaborated on, with the therapist ensuring that sufficient details of the memories are provided so as to promote increases in memory specificity. The respective therapeutic techniques are then utilized where appropriate, for example, restructuring beliefs about perceived failures and negative experiences, focusing on positive experiences and achievements to enhance perceptions of self-worth or self-efficacy, and breaking down previous coping step-by-step and highlighting effective strategies. As a general guideline, the therapist aims to help the individual create a cohesive and positive narrative of their life and a view of themselves that realistically integrates good and bad experiences. Further, therapists orient themselves towards improving clients’ perceptions of their meaning in life, self-esteem, self-efficacy, and optimism, given that these variables have been shown to mediate adaptive reminiscence functions and depressive symptoms [43] and changes produced by reminiscence-based therapies [44,45]. In the first session, a brief problem-solving framework is introduced, and a problem list is elicited for work in subsequent sessions. At the end of the session, a summary is provided and feedback is elicited from the participant on what was learned and any changes that have occurred. A homework sheet is then provided, listing questions related to the next week’s topic. A rationale is provided for this homework sheet, including its usefulness in promoting effective change [46]. The following five sessions follow a similar procedure of reviewing homework on the topic for the week, identifying memories to elaborate on and engaging in therapeutic work with these memories, and utilizing a problem-solving framework to choose solutions to identified problems to implement over the next week. Specific coping strategies that have been discussed in the session are drawn on to assist in the problem-solving work, as well as content related to self-efficacy and approach-coping behaviour that is broadly promoted over the sessions. In the last session, a summary is provided by the therapist of the work completed over the intervention, and the participant is invited to provide feedback of the experience and reflect on any change that has occurred.

Following the completion of the sixth session of therapy, participants will be free to utilize any further services or support that they wish. To ensure adherence to the treatment protocol, the treating therapist will complete a brief checklist following each session, noting whether required components of the therapy were covered in each session.

#### Usual care

Participants in the usual-care condition will receive support following the normal procedures implemented by the clinical pathway coordinator of the participating youth mental health service. There will be no restriction on the type, amount or length of support that is received. Given the service in which the study will be conducted, this support will probably consist of six to ten sessions of evidence-based psychological treatment provided by registered mental health professionals. Information will be collected for each participant on what their usual care constitutes so that it can be described as a group and contrasted with the cognitive-reminiscence group. Following the cessation of data collection, all participants in the usual-care group will be invited to receive the six-week course of cognitive-reminiscence therapy should they wish.

#### Outcome measures

All outcome measures will be completed online via links that are emailed to participants at the relevant time-points. As stated, the time-points will be baseline, three weeks into the intervention, post-intervention, one month post-intervention, and three months post-intervention. Multiple time-points will be used to increase statistical power [47], improve the chance of detecting when significant change occurs, and examine whether treatment effects are maintained following the end of treatment. The demographic information that will be collected from participants will be: age, sex, education, employment status, relationship status and whether or not previous treatment for depression has been received.

#### Primary outcomes

The primary outcome will be depressive symptomatology. To assess for depressive symptoms the seven-item self-report depression subscale from the short-form Depression, Anxiety, and Stress Scale (DASS-21 [38]) will be used. Each item is rated on a four-point scale from 0 (did not apply to me at all) to 3 (applied to me very much, or most of the time). The DASS-21 is a valid and reliable method of assessing core depressive symptomatology (low positive-affect, anhedonia, lack of motivation, low self-esteem and hopelessness), possesses good psychometric properties [48], and has been validated for use with young adults [49].

#### Secondary outcome measures

Recent meta-analytic results have indicated improvements across a number of domains of psychological wellbeing as a result of reminiscence-based therapy [15]. To assess the generalizability of these changes to young adults some of these will be used as secondary outcome variables. Across the following measures, participants will respond to 11-point, end-defined scales. Meaning in life will be measured using the presence subscale of the Meaning in Life Questionnaire [49], which comprises five items and has good psychometric properties [50]. Higher scores indicate a stronger sense of personal meaning. Self-efficacy will be measured using the eight-item New General Self-Efficacy Scale [51]. This scale measures general perceived competence underlying task or domain-specific self-efficacy using eight items, whereby higher scores indicate a stronger sense of general self-efficacy. The New General Self-Efficacy Scale is psychometrically strong, and has demonstrated superiority over other measures of general self-efficacy [52]. Self-esteem will be measured using a short-form, five-item version of the Rosenberg Self-Esteem Scale [53]. This short-form version has good internal reliability [42], and retains the measurement fidelity of the full scale [54]. Higher scores on this scale indicate higher levels of perceived self-worth.

Two additional secondary outcome measures will be assessed. Firstly, given the therapeutic aims of this intervention, it is reasoned that increases in optimism and positive future-orientation may be possible outcomes. To assess for this, a short-form, three-item version of the Life Orientation Test – Revised [55], consisting of the three positively worded items will be used. This test has demonstrated good psychometric properties [55], with high internal reliability also reported for the short-form version [43]. Higher scores on this scale indicate more positive generalized outcome expectancies. Secondly, as some previous trials of reminiscence-based therapies have also been shown to produce significant reductions in anxiety symptoms [56,57], this will also be assessed. The anxiety subscale on the DASS-21, which measures core symptoms of autonomic arousal, will be used to assess anxiety. The anxiety subscale of the DASS-21 shares the same scale design as the depression subscale, and also its sound psychometric properties.

#### Qualitative data

In addition to collecting demographics and outcome measures, one-to-one interviews with participants from the cognitive-reminiscence condition will also be conducted following completion of the final session of therapy. As the use of a reminiscence-based therapy is somewhat novel in young adult populations, the addition of qualitative data will provide richer, more detailed information regarding participants’ phenomenological account of the intervention. A semistructured interview schedule will be used to collect in-depth information on participants’ experiences. Open-ended questions with inductive probing will be used to answer research questions, such as what participants’ experience of the intervention was like, how they perceived the main task of discussing past events and experiences in their lives, which aspects they found helpful or unhelpful, whether the intervention produced any changes for them, their attitude towards the length of the intervention, and what they might like to see included that was not. To reduce the potential for bias, the interviews will be conducted by a researcher outside of the research team responsible for the trial. Interviews will be conducted with participants until data saturation is reached, or all willing participants have been interviewed.

#### Analysis

Baseline characteristics (demographic, clinical and wellbeing variables) will be compared using chi-square analyses for categorical variables and *t* tests for continuous variables. For the primary and secondary outcomes, repeated-measures ANOVAs will be conducted, along with appropriate post-hoc analyses. Effect sizes will be calculated using Cohen’s *d*, which is based on the pooled standard deviation, along with 95% confidence intervals. To assess individuals for clinically significant change on the primary outcome and distinguish between improved, unchanged and deteriorated cases, the reliable change index will be used [58]. Analyses will be conducted on post-treatment data using intention-to-treat methods presented in the CONSORT guidelines [59], with appropriate imputation of missing data. Qualitative data will be analyzed using applied thematic analysis techniques [60]. Interviews will be transcribed, coded and grouped into themes using an iterative process, and then the themes will be refined and interpreted. Quantitative data reduction techniques will also be used to analyze the frequency of particular attitudes and agreement or disagreement with specific questions, such as whether participants thought the intervention was long enough.

#### Consent and ethics

All participating young adults will be provided with a plain language statement, and receive a verbal explanation of its contents along with assistance in answering any questions they might have. They will then provide written informed consent to participate in the study. The project has been approved by the Deakin University Human Research Ethics Committee (DU-HREC 2012–278).

## Discussion

Depression is a prevalent problem for young adults, and is associated with a range of adverse outcomes, including impairments in social, occupational and physical functioning [61], increased experience of pain [62], and higher rates of mortality caused by an increased risk of suicide and physiological changes, such as immunosuppression that increase susceptibility to death [63]. The high economic burden of depression has been noted across a large number of studies [64] and is predominately related to such factors as loss of work productivity, increased cost of healthcare and specialist treatment [64,65]. Given this, as well as the fact that nonresponders to treatment are found across all treatment modalities and therefore no one treatment for depression is useful for all, further development of brief, effective treatments is justified.

This trial will help to evaluate a therapy among young adults that has a demonstrated record of large, clinically significant effects on depression in older adults. Given that young adults, relative to older adults, have a higher reported use of reminiscence for identity cohesion and clarity [66], and draw on the past to assist in task-based behaviour and clarify future goals [67], therapies such as cognitive-reminiscence therapy may be highly relevant for them. Quantitative and qualitative data gathered through this study will also help elucidate how and why this type of intervention might be helpful for young adults. These findings will assist in identifying changes that may need to be made to help further refine this therapy, and increase its specificity.

Despite the new findings that this trial is likely to generate, several limitations may be forecast. Perhaps most importantly, the small sample size of this trial is likely to limit the generalizability of the findings, and to limit the opportunity to identify moderating variables. However, as no pilot studies in this age group exist, and therefore the effectiveness of this therapy with young adults is largely unknown, a larger trial and the resources necessary to conduct it could not be justified at this stage. Depending on the outcomes of this trial, future research may look to utilize a larger sample. There is a chance that young adults will not respond well to this retrospectively oriented treatment modality, although no reliable evidence currently exists to support this contention, and a reasoned argument to the contrary has been constructed based on developmental and cognitive-based research [29].

In summary, the expected outcomes of this study are the validation of a manualized, therapeutic approach to treatment of depression in young adults, and new data concerning young adults’ experience of receiving this intervention and changes relating to broader psychological wellbeing that may occur. This manualized psychotherapy may then be implemented in mental health services to address the needs of young adults presenting with depressive symptoms.

## Trial status

Recruitment for the trial began in January 2013.

## Abbreviations

ANOVA: Analysis of variance; DASS: Depression, Anxiety, and Stress Scale.

## Competing interests

The authors declare that they have no competing interests.

## Authors’ contributions

DJH and DM conceived the project. DH produced the first and subsequent drafts of this paper, which DM reviewed. Both authors read and approved the final manuscript.
